# The importance of context—a qualitative study exploring healthcare practitioners’ experiences of working with patients at home after a stroke

**DOI:** 10.1186/s12913-023-09735-7

**Published:** 2023-07-06

**Authors:** Marie Elf, Dara Rasoal, Magnus Zingmark, Maya Kylén

**Affiliations:** 1 School of Health and Welfare, Dalarna University, Falun, Sweden; 2 Department of Health Sciences, Lund University, Lund, Sweden; 3 Health and Social Care Administration, Östersund, Sweden; 4 Department of Epidemiology and Global Health, Faculty of Medicine, Umeå University, Umåe, Sweden

**Keywords:** Rehabilitation, ESD, Built environment, Person-environment fit, Person-centred care, Life-space

## Abstract

**Background:**

Stroke significantly impacts individuals, leading to the need for long-lasting rehabilitation and adaptation to environmental demands. Rehabilitation after stroke is increasingly performed in patients’ homes, and it is argued that rehabilitation in this context is more person-centred and positively impacts client outcomes. However, the role of environmental factors in this process is largely unknown. The aim of this study was to explore how multidisciplinary healthcare practitioners working with rehabilitation in the home after stroke consider possibilities and challenges in the environment and how environmental factors are documented in patients’ records.

**Methods:**

Eight multidisciplinary healthcare practitioners working with home-based rehabilitation after stroke participated in two semistructured focus group sessions. Thematic analysis was used to analyse the transcripts of recorded focus group discussions. Data were also collected from patient history records (*N* = 14) to identify interventions to increase patients’ opportunities to participate in activities inside and outside the home. These records were analysed using life-space mobility as a conceptual framework.

**Results:**

The analysis generated four overarching themes concerning possibilities and challenges in the environment: (1) *the image of rehabilitation conflicts with place,* (2) *the person in the home reveals individual needs and capabilities,* (3) *environmental characteristics influence the rehabilitation practice,* and (4) *the person is integrated within a social context.* The patient record analysis showed that most patients were discharged from hospital to home within four days. Assessments at the hospital mainly focused on basic activities of daily living, such as the patient’s self-care and walking ability. Also at home, the assessments and actions primarily focused on basic activities with little focus on participation in meaningful activities performed in different life situations outside the home.

**Conclusions:**

Our research suggests that one way to improve practice is to include the environment in the rehabilitation and consider the person´s life space. Interventions should focus on supporting out-of-home mobility and activities as part of person-centred stroke rehabilitation. This must be supported by clear documentation in the patient records to strengthen clinical practice as well as the communication between stakeholders.

## Background

Stroke is a common disabling condition [1] that affects not only a person’s physical, cognitive, and emotional functions but also their identity, roles, and participation [2–4] and social isolation is common [5, 6]. Many face challenges related to mobility [7] and studies have shown that life-space mobility, conceptualized as movement patterns, is often restricted to the home or immediate surroundings [8, 9]. This is determined not only by physical and cognitive functioning but also by environmental factors [10], making it essential for healthcare practitioners to consider both supportive and hindering environmental factors in their clinical practice.

One of the goals of poststroke rehabilitation is to support patients in engaging in personally meaningful activities, living independently, and participating in society [11]. However, six months poststroke, 50% of persons lack meaningful activity [12]. Resumes to valued activities depends on the degree of disability and the environmental features in the home and close surroundings [13]. Nevertheless, it remains unclear how healthcare practitioners working with home-based rehabilitation relate to and document opportunities and challenges in the environment [14].

Home-based rehabilitation for people with stroke is an increasingly important way of responding to the population's need for integrated care [15]. Early supported discharge (ESD) is one service that aims to support the transfer of stroke care from the hospital to continued rehabilitation in the person’s familiar environment [16]. It is a multidisciplinary team intervention starting at the hospital and continues with stroke specific rehabilitation at home. The team consists of occupational therapists, physiotherapists, and often nurses and speech and language therapists [17]. ESD facilitates the persons involvement in the rehabilitation process and includes training activities at home but also outside the home, such as going shopping, doing laundry in a communal laundry room, or taking the bus [17]. ESD is recommended as standard care for persons with mild to moderate stroke as it reduces the number of in-hospital days and improves functional outcomes [18]. Nevertheless, the implementation process is slow [19], and the ways ESD teams are currently composed and practised vary widely [20]. In addition, even though stroke guidelines state that healthcare practitioners should focus on person-centred work and include the home environment as part of the rehabilitation process [21], persons with stroke have reported that the service is generic and that the environment is not sufficiently considered to support their recovery after stroke [22].

Earlier studies have shown that practitioners experience more flexibility and the ability to focus on people’s needs when rehabilitation occurs at home rather than in institutional care [23]. However, involvement in patients’ life situations can also be experienced as challenging concerning finding a balance between professionalism and privacy [24]. In addition, practitioners have noted that the home’s design, such as small bedrooms and bathrooms, can hinder rehabilitation activities [25]. Thus, providing care and rehabilitation in the home is complex [21], even more so now that hospital stays are short and the new standard is continued rehabilitation in the home [15], even for those with severe conditions.

International Classification of Functioning, Disability and Health (ICF) framework [26] highlights the impact of the context, including personal, social and environmental factors for health and well-being. ICF aligns well with the Ecological Theory of Aging (ETA) [27], which depicts that a person’s behaviour results from a dynamic relationship between their cognitive and physical competence and the demands in the environment. Negative outcomes may occur when the demands in the environment are too high or too low relative to a person´s functioning. For example, physical environment barriers such as stairs, uneven sidewalks can hinder a person's ability to function at home and limit opportunities to participate in community activities [28, 29]. This underscores the importance of housing adaptations for people with stroke after discharge from the hospital [30] and incorporating environmental factors into rehabilitation since it may have significant consequences for meeting people's rehabilitation needs. Documenting relevant factors and interventions in patient records is crucial for ensuring the continuity of care. Such documentation is an integral component of holistic patient care and rehabilitation while facilitating a comprehensive understanding of the patient's unique contextual factors. The patient record serves as a collaborative tool for the healthcare team, facilitating care coordination and rehabilitation. It ensures compliance with legal requirements by documenting planned and completed interventions, while also enabling effective communication among team members. Insufficient information in the patient's medical record can hinder communication between staff and the various stakeholders providing care and thus influence the therapeutic process [31]. However, empirical data on how environmental factors are documented in stroke practice are currently lacking.

In this study, we explored how multidisciplinary healthcare practitioners working with home rehabilitation after stroke reason about possibilities and challenges in the environment and how environmental factors are documented in patients’ medical records. The research questions and data triangulation can provide a comprehensive understanding of the environment's importance in rehabilitation at home [32]. Thus, the following research questions were addressed:

I How is the environment used and considered by healthcare practitioners in rehabilitation at home?
II What are the perceived challenges and opportunities?
III How is the environment documented in patients’ records?

## Methods

We used an explorative qualitative design using focus group (FG) methodology and patient record analysis. To answer research questions I and II, we recruited eight practitioners from three hospitals in southern Sweden working with home-based stroke rehabilitation to participate in two FG. The three hospitals were chosen as the research team had contacts within the management in southern Sweden. None of the researchers knew the participants from before. One of the key strengths of the FG as a method is the synergy [33] created in a group context, which generates momentum and allows opinions, beliefs, feelings, and attitudes to emerge in parallel with individual experiences [34]. The participants were recruited based on similarities (homogeneity criteria), which were dictated by the purpose of the study, and differences (heterogeneity criteria) between them to explore different perspectives [34]. The participants were interested in discussing possibilities and challenges in the patients' home environments (homogeneity criterion). Heterogeneity among the participants was achieved through diverse ages, types of professions (e.g., occupational therapist, physiotherapist, nurse) and years of working with home rehabilitation.

The interview guide (see Table 1 for topics included) was developed based on findings from our previous research with stroke patients [22, 35], theories underpinning the project, and relevant literature. Using the preliminary results from the first session, we adapted the interview guide for the second session to explore the constructed themes.

**Table 1:** Focus group topics

Opening question: *Tell us your name, what you work with and what is most fun about your work*
*What is important to consider in the environment when it comes to rehabilitation at home?* *• How/in what way do you use the environment in the home/local area for rehabilitation purposes?* *• How is the environment considered in planning/follow-up of rehabilitative interventions?* *• How is the environment used in the implementation of rehabilitation?* *• What difficulties/opportunities do you experience with rehabilitation at home?*
*What is the basis for how you plan rehabilitation at home?* *• How is the patient included in the planning?*
*How is the patient involved in the actual implementation of the rehabilitation?* *• What is important regarding participation in rehabilitation at home?*

Two moderators led each group: one led the discussions, and the other observed group interactions and listened to the discussions. To accommodate the practitioners’ work schedules, sessions were held online, and audio recorded. Sessions lasted approximately 1.5 h. The observing moderator took field notes on group dynamics and posed clarifying questions.

To answer research question III, copies of electronic patient records (*N* = 14) were obtained from the different care contexts after each patient’s rehabilitation period. The patient records were entirely anonymized before the analysis. This process entailed removing or replacing the names of patients and healthcare practitioners, dates of birth, and addresses with a number. The data were securely stored according to data protection regulations at Dalarna University.

### Analysis

In line with Braun and Clarke, the data analysis for the FG discussions was inspired by reflexive thematic analysis underpinned by a combination of critical theory and realism [36]. Such approach acknowledges that social reality is shaped by both objective structures and subjective interpretations [37]. Hence, seeking to explore how multidisciplinary healthcare practitioners’ reason about possibilities and challenges in the environment we did not only set out to identify and describe (fundamental to simple realism) but also to critically examine the social context and underlying processes that contribute to the construction and interpretation of themes [37]. We used NVivo software to document the analytical process [38] which was conducted in several iterative phases as described below. Acknowledging that each individual researcher´s previous experiences and preconceptions may influence the way data is interpreted peer-debriefing meetings were held throughout the analysis process to discuss codes and potential own biases.

First, we (co-authors DR and MK) familiarized ourselves with the data by reading and rereading the transcripts. Second, text that was relevant to the research aim was highlighted and segments of data was coded. Third the research team (all co-authors) reviewed, compared, and discussed the codes. Codes were grouped based on similarities and differences and merged into subcategories and themes. Alternative explanations were constantly sought for, and a reflexive journal was kept in NVivo to document thoughts, interpretations, and decisions. In a last phase, the subcategories and themes were discussed again among the authors until a consensus was reached.

**Fig. 1:**
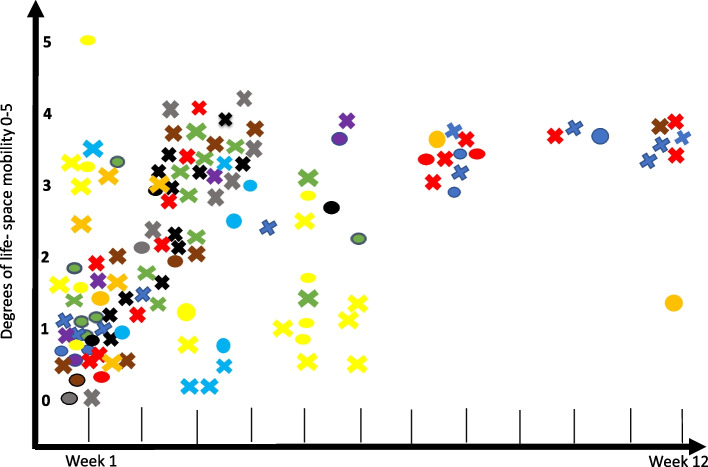
Information about the environment and a person’s life-space zones (0 bedroom, 1 the rest of the house, 2 the very immediate surroundings, 3 the local community or neighbourhood, 4 the town, and 5 unlimited areas) throughout the care chain. Circles represent medical history taking, and crosses involve assessment, interventions, and follow-up. Each colour represents one case (*N* = 14)

To analyse the patient records and identify if and how interventions to increase patients’ opportunities to participate in activities inside and outside the home were documented, we used life-space mobility (LSM) as a conceptual framework. LSM is structured into spatial zones and reveals the frequency and independence (i.e., need for assistance from another person/mobility aids) of a person’s movements across life-space zones over a given period [39]. The five life-space zones include 0) bedrooms, followed by activities performed in 1) the rest of the house, 2) the very immediate surroundings (e.g., garden), 3) the local community or neighbourhood, 4) the town, and 5) unlimited areas. Based on this, we created a coding scheme, and two of the authors (MK and DR) extracted all documented information regarding the environment as information either in a patient history format (circle) or as an assessment, intervention, or follow-up (cross). This information was later plotted in a diagram with life-space zones on the y-axis and time on the x-axis (Fig. 1). Interventions aiming to visit a rehab centre, or some other type of healthcare environment were excluded from the figure.

## Results

Eight practitioners (all women) from three hospitals in southern Sweden participated in the two FG sessions. The practitioners (four occupational therapists, three physiotherapists, and one nurse) had experience working with home rehabilitation after stroke ranging from 10 months to 7 years. The analysis generated four overarching themes: (1) *the image of rehabilitation conflicts with place,* (2) *the person in the home reveals individual needs and capabilities,* (3) *environmental characteristics influence the rehabilitation practice,* and (4) *the person is integrated within a social context* (Table 2)*.*

**Table 2:** Summary of key themes and subcategories identified in the FG discussions

The image of rehabilitation conflicts with place	The person in the home reveals individual needs and capabilities	Environmental characteristics influence the rehabilitation practice	The person is integrated within a social context
*Confronting the view of the institution as the best place for rehabilitation*	*The person in the home makes it easier to set meaningful goals*	*One home is never the same as another*	*Additional actors and a shift in power and roles*
*Meeting relative’s needs and uncertainties*	*The safe home as a point of departure boosts the rehabilitation*		
	*Too fast or too slow – the importance of balance*		

### The image of rehabilitation conflicts with place

#### Confronting the view of the institution as the best place for rehabilitation

The practitioners stated that they often had challenging discussions with patients about the best place to rehabilitate before discharge from the hospital. Some patients easily agreed with the plan for continued rehabilitation at home, while others had different preferences (e.g., rehabilitation unit/home) and rehabilitation content. Conflicts could occur when the place for rehabilitation was the opposite of the patient’s expressed preferences.

*“Yes, I thought about this with what the patient wants and how to sort of get over it because it can be that you give them different options for the type of rehabilitation after discharge and then... maybe they still say yes, but I want to go to XXX (rehab service centre for inpatients). Even though we may not always agree, they must still be allowed to…. express their wish.”* (Occupational therapist 1, ESD 14 moths of experience, hospital 1) 1

Such a situation could lead to negotiations with a patient with the aim of getting the patient to accept continuing rehabilitation at home after inpatient care. Sometimes patients did not want help from the practitioners, which was a challenge because, from their professional experience, they could see that many needed continued support.

*“I can practice myself; I do not need it (rehabilitation at home), they say. And sometimes it can be the case that we disagree with that view, but we cannot force ourselves on them; we can just give different suggestions.”* (Occupational therapist 2, ESD 14 moths of experience, hospital 1)

Even though rehabilitation at home was perceived as very positive for both the patient and the practitioner, it also entailed the challenge of making the patient understand the home as a place for rehabilitation.

*“Many ask about….training and then….something that we talk a lot about with our patients is focused activity training. It (the question) comes from all ages. Many people think about what we are going to do with them at home…it is probably 95% who ask that question before we have explained the importance of everyday activities and that we use everything that is around them, things they usually have done before…so the focus is quite strong on that particular aspect.”* (Occupational therapist, ESD 3 years of experience, hospital 2)

Specifically, the practitioners noted that it was difficult to make the patients understand how objects and environmental features in their home and close surroundings could facilitate training of different body functions, such as fine motor skills and cognition. The practitioners often needed to give direct feedback after a specific activity to achieve a shared understanding. By telling patients, for example, that they had trained their fine motor skills and balance, the patients understood the value of these everyday activities for their recovery.

#### Meeting relatives’ needs and uncertainties

Sometimes relatives influenced the patient’s wish to be rehabilitated at home by not accepting home-based rehabilitation. Instead, they wanted to move the patient into a rehabilitation facility. As explained by the practitioners, one reason for this was that the relatives felt too much responsibility.

*“Many times, I think, you have to have that discussion with the relatives because…they insist and want the patient to go (to the rehabilitation facility) because they feel that it is too big of a responsibility forced on them.”* (Physiotherapist, ESD 7 years of experience, hospital 2)

Relatives often believed that the rehabilitation facility would create "miracles.” At the same time, the practitioners emphasized that relatives were often shocked by the whole situation and needed support. To make relatives understand the rehabilitation process, they carefully explained the patients’ limitations or challenges.

*“You must explain (to the relatives) both before and after what limitations or challenges the individual has and what they have done before (activity level).”* (Occupational therapist, ESD 3 years’ experience, hospital 2).

### The person in the home reveals individual needs and capabilities

#### The person in the home makes it easier to set meaningful goals

In the home, seeing how the person interacts with the environment facilitates a better understanding of the person’s abilities, limitations, and individual needs. In the standardized hospital environment, it is difficult to discover where in the recovery process a person is.

*“It becomes more real when you are in the home environment, and it is better.... easier to communicate, [… ] they see the same thing as I see, because I do not see what they described when they were in the hospital ward.”* (Physiotherapist, ESD 3 years’ experience, hospital 3)

Being at home made it easier to provide person-centred rehabilitation as they could focus more on matters that were truly important to the patient and use those activities as an opportunity for training. This improved communication as they (healthcare practitioner and patient) focused on the same thing.

*“It is much easier, as we have said before, getting yourself focused on what is most important for the particular patient.”* (Occupational therapist 2, ESD 14 moths of experience, hospital 1)

Goals for rehabilitation were formed with the patient based on the activities performed. For example, by engaging in valued activities in the garden related to the patients´ goals, motor functions cold be trained. Some practitioners used established goal-setting instruments to guide their rehabilitation planning. They focused on what motivated the patient, which was vital for a successful rehabilitation process.

*“We usually start and set goals fairly immediately, and then it is the patient's motivation that directs what he or she wants to do, and you can use this instrument fairly clearly.”* (Occupational therapist, ESD 10 moths of experience, hospital 3)

The practitioners also described how they had to be flexible, adapt and perform rehabilitation activities differently depending on the unique characteristics of the person’s home environment. Given the large variety of home environments, they could not follow a standardized rehabilitation procedure but rather tailored the interventions to fit each patient’s environment.

#### The safe home as a point of departure boosts the rehabilitation

The home as a safe place emerged as a central topic. The practitioners mentioned that the patient’s emotional bonds and feelings of belonging to home facilitated rehabilitation and supported the patient’s recovery after stroke. Thus, the home rehabilitation format was described as holistic, and practitioners could support the patient in ways that were impossible in the hospital.

*“And then it's not just physiotherapy and occupational therapy, but it's like… it's a little more than that for us…if you should need and cannot come to the pharmacy and buy, for example, incontinence protection, then I can call or bring it with me the next time I come. So, it becomes… it's the whole.”* (Nurse, ESD 3,5 years of experience, hospital 3)

*“It is almost only positive that I can come home to them, and they (the patient) can feel safe in this and get support in this. It is…it is usually just positive.”* (Occupational therapist, ESD 10 moths of experience, hospital 3) 

### Too fast or too slow – the importance of balance

The practitioners emphasized that they often had to advise the patients not to hurry with their rehabilitation and overwork themselves. They wanted to protect the patients as they knew that stress could result in more severe brain fatigue or interfere with social relationships.

*“Many persons are...in such a hurry… and we try to make them understand that brain fatigue can have an impact on the first stage of their rehabilitation.”* (Occupational therapist, ESD 3 years of experience, hospital 2)

In contrast, some patients had a low activity level because of their physical or cognitive limitations or because they were afraid to fall and hurt themselves when doing tasks at home independently. This was described as challenging by the practitioners, who tried to encourage patients to achieve further goals. Sometimes it could also be challenging to get patients active in their homes as they focused on obstacles.

### Environmental characteristics influence the rehabilitation practice

#### One home is never the same as another

Even if the practitioners asked the patient at the hospital about his or her environment at home, it was when they first met the person in the home that they became aware of, for example, environmental obstacles such as thresholds, stairs, or limited spaces. This was common and could lead to interventions involving the removal of furniture or starting a housing adaptation process.

In some dwellings, it was easier to refurnish objects such as chairs, bookshelves, beds, or equipment in the kitchen. However, permanent housing adaptations were more difficult. On the other hand, obstacles in the home could be used as part of the rehabilitation and as a goal for a patient to achieve instead of adapting to the environment.

*“The environment can both facilitate and cause problems; for example, we had a man who could not get up to where he had his bed. It was almost impossible to get up there, so he had to sleep at a different place just because he could not go up the stairs. So that became an obvious goal for him: to manage the stairs. So yes, there are both advantages and disadvantages.”* (Occupational therapist 2, ESD 14 moths of experience, hospital 1)

The home also facilitated creativity and offered many more opportunities and rehabilitation tools than at the hospital.

*“It (the home) is a whole smorgasbord with all the things that person usually does, so that …it makes it easier to focus on those activities that are most important for the individual patient.”* (Physiotherapist, ESD 3 years’ experience, hospital 3)

### The person is integrated within a social context

#### Additional actors and a shift in power and roles

The practitioners said they needed to consider the patient’s integration in a social context. For example, gender roles assign women the role of carers, which was experienced as a considerable challenge.

*“But I have to say, aspects such as cultural, social, relatives…this part makes home rehabilitation a little more complicated. Here in XXX (name of the city), we have a grand mix of clients, which makes a big difference, and it is a huge challenge.”* (Occupational therapist, ESD 3 years of experience, hospital 2)

After a stroke, family roles are often changed, requiring special attention from practitioners. For example, performing daily chores such as doing laundry or cooking food may be complex, meaning that someone in the family needs to take over and be responsible for such activities.

The practitioners also noted the power shift that occurs when they enter a patient’s home. At the hospital, they had a straightforward routine to follow, and the conditions and atmosphere gave them specific power to act. At home, the patient was described as more empowered and had a stronger voice than in the hospital, which the practitioners needed to respect and consider.

The practitioners also needed to consider the patient’s needs regarding other family members’ wishes. Patients and their family members did not always agree with the practitioners’ suggestions, which could be challenging.

*“Then you are inside… another person’s home and the family, so… of course you have to be a little careful when you start, before you know who it is you have to deal with, so to speak.”* (Physiotherapist, ESD 3 years’ experience, hospital 3)

## Results from the patient record analysis

The patient record analysis (*N* = 14 patients) revealed that the hospital stay after a stroke was short. Most patients were discharged home within four days. At the hospital, patient history taking included asking whether the patient lived alone or with someone, the type of housing, and the possibilities for entering and exiting (LSM 0–2). Assessments and actions at the hospital mainly focused on basic ADL activities, such as the patient’s ability for self-care and walking indoors (LSM 0–1). After discharge, interventions were limited both with the number of contacts and durations. At home, the assessments, actions and follow-ups primarily focused on the same basic ADL activities as in the hospital. As seen in Fig. 1, few interventions aimed to help the patient be active in society.

The lack of interventions promoting out-of-home mobility can be exemplified by the following record (duration 6 weeks). It was noted in the medical history taking that (due to mobility limitations and no support from family) “*the patient is completely cut off from all activities outside the home*” (yellow circle in the higher degree of life-space in Fig. 1).

The same patient record noted that the person lived in a socially deprived area with “*no lock on the front door,*” and the environment in and around the apartment was described as “*dirty*.” However, the interventions to support this patient were related to personal hygiene, namely, prescribing technical aids to assist with showers and getting up from the toilet (yellow cross representing the intervention and follow-up on the x-axis in Fig. 1).

## Discussion

Our study focused on how multidisciplinary healthcare practitioners work with home rehabilitation after stroke and how they perceive possibilities and challenges in the environment, and how environmental factors are documented in patients’ records. The key contribution is the unique focus on how the environment is used in rehabilitation, underpinned by the robustness of triangulated data [32]. The findings illustrate a complex situation involving many actors and environments requiring specific competencies among practitioners. While the FG participants expressed how physical and social environmental aspects significantly impacted the whole context and opportunities for home rehabilitation, this was not evident in the analysis of patient records. For example, we found that few interventions to support out-of-home activities were documented. Most entailed help to visit a rehab centre or some other type of healthcare setting. This finding deserves attention as the home and neighbourhood can provide a central starting point for identifying prioritized activities and setting person-centred goals, as shown in a Norwegian reablement context [40]. Constrained life-space coexists with a loss of meaningful activities (e.g., participating in out-of-home activities, recreational activities) and participation restrictions in terms of the inability to access community resources and services [41–43], which in turn increases the risk for depressive symptoms [44]. With such knowledge, healthcare practitioners should support patients in maintaining their life-space and engaging in meaningful activities both at home and outside the home.

The findings from the patient records are important and worth sharing for further research. However, investigating how the environment was documented was a challenging task. Due to different IT systems across the care pathway, the patient records took time to identify. This was especially true for patients with several healthcare providers simultaneously. In addition, finding the information necessary to answer the research questions was sometimes challenging. Thus, the current IT-system design used in Swedish healthcare may provide limited opportunities to document environmental factors and follow the documentation between different healthcare providers.

The findings from the FG showed that the practitioners appreciated working with home-based rehabilitation as it was flexible and facilitated person-centred care and goal setting. This was also reported in another qualitative study showing that practitioners used the home environment in their poststroke interventions to support the patient in returning to everyday life [45]. However, based on our findings, we cannot say to what extent the rehabilitation was structured as a person-centred intervention. While the practitioners said that the home environment contributed to a shared understanding of abilities, limitations, and individual needs that facilitated goal setting, they also stated that the patients had difficulties understanding when they were training body functions. This could mean that practitioners do not sufficiently include the patient in rehabilitation goal setting. This is an area for improvement since previous research has found that using a structured process for goal identification may be effective and improve health outcomes [46].

In addition, the practitioners in our study revealed that the patient’s image of where the rehabilitation should occur sometimes did not correspond with the home. The difficulty of communicating about goals and what the environment can contribute to the rehabilitation was discussed in another study focusing on stroke patients’ experiences of home rehabilitation [22]. This underlines the importance of communication to obtain a shared understanding of the goal of rehabilitation and suggests that it can be conducted with good results in a natural environment such as the home.

In line with other studies [47, 48], the practitioners acknowledged that the home is closely related to identity and that being at home supported the patients’ recovery. Dimensions of the home include emotional, cognitive (e.g., conveying "identity"), behavioural, social, and physical dimensions [49], which people may experience differently after a stroke [47]. Currently, when care and rehabilitation increasingly occur at home, practitioners must consider all these dimensions and involve the person in the rehabilitation planning process, particularly for persons with long-term conditions.

The finding that addressing relatives’ needs and uncertainties is part of rehabilitation at home is confirmed by other studies. For example, Hjelle and colleagues [50] found that relatives often wish to contribute, but at the same time, they do not want to engage too much as the caring and supporting tasks becomes burdensome. The social participation of relatives after a stroke has been characterized by expanded obligations and a reduction in social relationships and leisure activities [51], which is essential to consider. The practitioners in our study emphasized that relatives were often shocked by the situation and felt a need to support them. This is a complex situation and focusing on out-of-home activities may promote health and wellbeing for patients and their relatives [52]. Future research should focus on developing approaches to support practitioners in involving relatives as part of rehabilitation at home and helping them in their situation.

Our participants described how goals for rehabilitation were formed together with the patient and based on activities performed in the patient’s home. However, while insight into each person’s goals and preferences in rehabilitation is crucial to tailor each intervention individually, the focus on activities performed inside of the home might be limiting for some persons. From a patient perspective, a study found that patients prioritize functional mobility indoors and outdoors for rehabilitation [40]. Nevertheless, persons with stroke have described that few interventions focus on outdoor activities and their desire to be part of society [22]. Therefore, while the in-home environment is vital for many persons after a stroke, it is also essential to include out-of-home environments as a potential focus for rehabilitation activities.

In their study on older adults receiving home-based services, Vik and Eide [53] found that indoor mobility, personal ADL, and leisure activities were considered essential among older adults receiving home-based services. In contrast, participation in leisure and social activities were areas of life with which they were least satisfied. A Swedish survey study investigating occupational therapy and physiotherapy interventions revealed a strong focus on indoor mobility and personal ADL. In contrast, outdoor mobility and social participation were scarcely addressed [54]. These findings are hardly surprising. Rehabilitation has traditionally focused on improving people's bodily functions [5] rather than supporting people in participating in society. However, according to stroke guidelines [55], the goal of rehabilitation should be to improve the person's activity and participation in society. Such an approach to rehabilitation would be well supported by previous research on what persons with stroke consider essential (e.g., [22, 56, 57]).

Additionally, we found that follow-up was not a usual practice, which might explain some of the findings. When a person comes home from the hospital, interventions naturally need to focus on indoor activities and basic ADL. However, this is only an initial part of rehabilitation, which should also focus on long-term goals such as participation in society. As suggested in another study [58], subsequent goals focusing on social participation and out-of-home activities should be established at follow-up.

### Limitations

All participants were women and were recruited from healthcare organizations in southern Sweden, which was a limitation in terms of heterogeneity. However, women are overrepresented in allied healthcare professions, and no men were working in the ESD teams from which we recruited participants. We received high-quality data from the practitioners. Yet, since home-based rehabilitation is increasing in all areas of healthcare, more studies are needed to understand different experiences and how they may differ depending on the healthcare context. The FG discussions were conducted online, which was limiting as it is easier to moderate a discussion when all participants are in person. Nevertheless, we found the approach beneficial as it was easier to arrange, less expensive, and less time consuming. With regard to the patient medical records, the patients included were not the same patients who had met the practitioners we interviewed, which was a limitation. However, they had been through the same care organization, meaning that the findings somewhat mirrored the current practice.

## Conclusion

Improving home-based healthcare for persons with complex health conditions is a primary policy objective. Our research suggests that one way to improve practice is to include the environment in rehabilitation and consider the person´s life space. Reduced mobility is a well-known impact of stroke, so is muscle weakness, falls and unmet needs related to ADL. This means that the demands in the environment are often too high and practitioners need to focus their interventions supporting patients in this respect. Importantly, follow-up should be routine practice, and when all goals are fulfilled, new goals focusing primarily on the out-of-home context should be established. To the best of our knowledge, our study is the first to use life-space mobility as a conceptual framework when analysing patient records. By doing so, we can identify contradictions between the practitioners’ narratives and what was documented. That is, the documentation lacks important components, which is a serious issue if the inadequate documentation mirrors the actual rehabilitation. Whether there is a need for improved IT systems or knowledge among practitioners is an issue for future research. Nevertheless, there is a need to develop interventions to address out-of-home mobility and activities as part of person-centred stroke rehabilitation.

## Data Availability

The datasets used and/or analyzed during the current study are available from the corresponding author on request.

## Footnotes

1 Two of the occupational therapists at hospital 1 started at the same time and are thus labeled 1 or 2.

## References

[1] Strong K, Mathers C, Bonita R (2007). Preventing stroke: saving lives around the world. Lancet Neurol.

[2] Hole E, Stubbs B, Roskell C, Soundy A. The patient’s experience of the psychosocial process that influences identity following stroke rehabilitation: a metaethnography. Sci World J. 2014;2014. 10.1155/2014/349151. 10.1155/2014/349151 PMC3927748 24616623

[3] Salter K, Hellings C, Foley N, Teasell R (2008). The experience of living with stroke: a qualitative meta-synthesis. J Rehabil Med.

[4] Nilsson I, Jansson L, Norberg A (1997). To meet with a stroke: patients’ experiences and aspects seen through a screen of crises. J Adv Nurs.

[5] Northcott S, Moss B, Harrison K, Hilari K (2016). A systematic review of the impact of stroke on social support and social networks: associated factors and patterns of change. Clin Rehabil.

[6] Jansen HE, Schepers VP, Visser-Meily JM, Post MW (2012). Social activity one and three years post-stroke. J Rehabil Med.

[7] Stephan KM, Pérennou D. Mobility After Stroke: Relearning to Walk. In: Platz T, editor. Clinical Pathways in Stroke Rehabilitation: Evidence-based Clinical Practice Recommendations. Cham (CH): Springer; 2021. p. 123–147. 36315701

[8] Tashiro H, Isho T, Takeda T, Nakamura T, Kozuka N, Hoshi F (2019). Life-space mobility and relevant factors in community-dwelling individuals with stroke in Japan: a cross-sectional study. Prog Rehabil Med.

[9] Hinrichs T, Rössler R, Infanger D, et al. Self-reported life-space mobility in the first year after ischemic stroke: longitudinal findings from the MOBITEC-Stroke project [published online ahead of print, 2023 May 4]. J Neurol. 2023;1–12. 10.1007/s00415-023-11748-5. 10.1007/s00415-023-11748-5 PMC10157571 37140729

[10] Webber SC, Porter MM, Menec VH (2010). Mobility in older adults: a comprehensive framework. Gerontologist.

[11] Norrving B, Barrick J, Davalos A, Dichgans M, Cordonnier C, Guekht A (2018). Action plan for stroke in Europe 2018–2030. Eur Stroke J.

[12] Mayo NE, Wood-Dauphinee S, Coˆte R, Durcan L, Carlton J (2002). Activity, participation, and quality of life 6 months poststroke. Arch Phys Med Rehabil.

[13] Della Vecchia C, Viprey M, Haesebaert J, Termoz A, Giroudon C, Dima A (2021). Contextual determinants of participation after stroke: a systematic review of quantitative and qualitative studies. Disabil Rehabil.

[14] Fisher RJ, Riley-Bennett F, Russell L, Lee C, Sturt R, Walker M, et al. Nominal group technique to establish the core components of home-based rehabilitation for survivors of stroke with severe disability. BMJ open. 2021;11(12):e052593. 10.1136/bmjopen-2021-052593. 10.1136/bmjopen-2021-052593 PMC8640659 34857570

[15] Swedish Government Official Reports (SOU). God och nära vård – En reform för ett hållbart hälso-och sjukvårdssystem. Report No: 2020:19: Swedish Government Official Reports; 2020. https://www.regeringen.se/contentassets/320f37078d854712ab89e8185466817b/god-och-nara-vard-en-reform-for-ett-hallbart-halso--och-sjukvardssystem-sou_2020_19_webb.pdf. Accessed 3 July 2023.

[16] Langhorne P, Baylan S, Trialists ESD. Early supported discharge services for people with acute stroke. Cochrane Database Syst Rev. 2017; 13;7(7):CD000443. 10.1002/14651858.CD000443.pub4. 10.1002/14651858.CD000443.pub4 PMC6483472 28703869

[17] Swedish Council on Health Technology Assessment. Rehabilitation at Home After Early Supported Discharge (ESD) for Elderly Patients After Stroke. Stockholm Report No. 234: Swedish Council on Health Technology Assessment (SBU); 2015. https://pubmed.ncbi.nlm.nih.gov/26803854/. Accessed 3 July 2023. 26803854

[18] Fearon P, Langhorne P, Trialists ESD. Services for reducing duration of hospital care for acute stroke patients. Cochrane Database Syst Rev. 2012;12;(9):CD000443. 10.1002/14651858.CD000443.pub3. 10.1002/14651858.CD000443.pub3 22972045

[19] Riksstroke. Stroke och Tia. Yearly report. 2021 September. Available from: https://www.riksstroke.org/sve/forskning-statistik-och-verksamhetsutveckling/rapporter/arsrapporter/.

[20] Mas MÀ, Inzitari M (2015). A critical review of Early Supported Discharge for stroke patients: from evidence to implementation into practice. Int J Stroke.

[21] von Koch L, Holmqvist LW, Wottrich AW, Tham K, de Pedro-Cuesta J (2000). Rehabilitation at home after stroke: a descriptive study of an individualized intervention. Clin Rehabil.

[22] Kylén M, Ytterberg C, von Koch L, Elf M (2021). How is the environment integrated into post-stroke rehabilitation? A qualitative study among community-dwelling persons with stroke who receive home rehabilitation in Sweden. Health Soc Care Community.

[23] van der Veen DJ, Döpp CM, Siemonsma PC, Nijhuis-van der Sanden MW, de Swart BJ, Steultjens EM. Factors influencing the implementation of home-based stroke rehabilitation: professionals’ perspective. PloS one. 2019;14(7):e0220226. 10.1371/journal.pone.0220226. 10.1371/journal.pone.0220226 PMC6657875 31344103

[24] Martinsen R, Kitzmüller G, Mangset M, Kvigne K, Evju AS, Bronken BA, et al. Nurses’ and occupational therapists’ experiences of conducting a home-based psychosocial intervention following stroke: a qualitative process evaluation. BMC Health Serv Res. 2021;21(1):1–10. https://link.springer.com/journal/12913. 10.1186/s12913-021-06857-8 PMC8356405 34376188

[25] Pettersson C, Nilsson M, Andersson M, Wijk H. The impact of the physical environment for caregiving in ordinary housing: Experiences of staff in home-and health-care services. Appl Ergon. 2021;92:103352. 10.1016/j.apergo.2020.103352. 10.1016/j.apergo.2020.103352 33395590

[26] Ustün TB, Chatterji S, Bickenbach J, Kostanjsek N, Schneider M (2003). The International Classification of Functioning, Disability and Health: a new tool for understanding disability and health. Disabil Rehabil.

[27] Lawton MP, Nahemow L. Ecology and the aging process. Washington, DC: American Psychological Association; 1973.

[28] Keysor JJ, Jette AM, Coster W, Bettger JP, Haley SM (2006). Association of environmental factors with levels of home and community participation in an adult rehabilitation cohort. Arch Phys Med Rehabil.

[29] Rochette JD, Luc Noreau, Annie. Association between personal and environmental factors and the occurrence of handicap situations following a stroke. Disabil Rehabil. 2001;23(13):559–69. 10.1080/09638280010022540. 10.1080/09638280010022540 11451190

[30] S⊘ rensen HV, Lendal S, Schultz-Larsen K, Uhrskov T. Stroke rehabilitation: assistive technology devices and environmental modifications following primary rehabilitation in hospital—a therapeutic perspective. Assist Technol. 2003;15(1):39–48. 10.1080/10400435.2003.10131888. 10.1080/10400435.2003.10131888 14760980

[31] McCarthy B, Fitzgerald S, O’Shea M, Condon C, Hartnett-Collins G, Clancy M (2019). Electronic nursing documentation interventions to promote or improve patient safety and quality care: A systematic review. J Nurs Manag.

[32] Lincoln YS, Guba EG. Establishing trustworthiness. In: Yvonna L, Egon G, editors. Naturalistic inquiry. Beverly Hills: Sage Publications; 1985. p. 289–327.

[33] Kitzinger J (1994). The methodology of focus groups: the importance of interaction between research participants. Sociol Health Illn..

[34] Parker A, Tritter J (2006). Focus group method and methodology: current practice and recent debate. Int J Res Method Educ.

[35] Kylén M, Von Koch L, Pessah-Rasmussen H, Marcheschi E, Ytterberg C, Heylighen A (2019). The Importance of the Built Environment in Person-Centred Rehabilitation at Home: Study Protocol. Int J Environ Health Res.

[36] Braun V, Clarke V (2006). Using thematic analysis in psychology. Qual Res.

[37] Braun V, Clarke V. Thematic analysis: A practical guide. Los Angeles: SAGE; 2022.

[38] Edhlund B, McDougall A. NVivo 12 essentials. Stallarholmen: Form & Kunskap; 2019.

[39] Baker PS, Bodner EV, Allman RM (2003). Measuring life-space mobility in community-dwelling older adults. J Am Geriatr Soc.

[40] Tuntland H, Kjeken I, Folkestad B, Førland O, Langeland E. Everyday occupations prioritised by older adults participating in reablement. A cross-sectional study. Scand J Occup Ther. 2020;27(4):248–58. 10.1080/11038128.2019.1604800. 10.1080/11038128.2019.1604800 31136214

[41] Brown CJ, Roth DL, Allman RM, Sawyer P, Ritchie CS, Roseman JM (2009). Trajectories of life-space mobility after hospitalization. Ann Intern Med.

[42] Byles JE, Leigh L, Vo K, Forder P, Curryer C (2015). Life space and mental health: a study of older community-dwelling persons in Australia. Aging Ment Health.

[43] Murata C, Kondo T, Tamakoshi K, Yatsuya H, Toyoshima H (2006). Factors associated with life space among community-living rural elders in Japan. Public Health Nurs.

[44] Polku H, Mikkola TM, Portegijs E, Rantakokko M, Kokko K, Kauppinen M (2015). Life-space mobility and dimensions of depressive symptoms among community-dwelling older adults. Aging Ment Health.

[45] Wottrich AW, von Koch L, Tham K (2007). The Meaning of Rehabilitation in the Home Environment After Acute Stroke From the Perspective of a Multiprofessional Team. Phys Ther.

[46] Parsons J, Rouse P, Robinson EM, Sheridan N, Connolly MJ (2012). Goal setting as a feature of homecare services for older people: does it make a difference?. Age Ageing.

[47] Hodson T, Aplin T, Gustafsson L (2016). Understanding the dimensions of home for people returning home post stroke rehabilitation. Br J Occup Ther.

[48] Meijering L, Nanninga CS, Lettinga AT. Home-making after stroke. A qualitative study among Dutch stroke survivors. Health Place. 2016;37:35–42. 10.1016/j.healthplace.2015.11.006. 10.1016/j.healthplace.2015.11.006 26702961

[49] Oswald F, Wahl H-W. Dimensions of the meaning of home in later life. In: Rowles G, Chaudhury H, editors. Home and identity in late life: International perspectives. New York: Springer Publishing Company, Inc.; 2005. p.21–45.

[50] Hjelle KM, Alvsvåg H, Førland O. The relatives’ voice: how do relatives experience participation in reablement? A qualitative study. J Multidiscip Healthc. 2017;10:1. 10.2147/FJMDH.S122385. 10.2147/JMDH.S122385 PMC5207447 28096681

[51] Pellerin C, Rochette A, Racine E (2011). Social participation of relatives post-stroke: the role of rehabilitation and related ethical issues. Disabil Rehabil.

[52] Nyman A, Josephsson S, Isaksson G (2014). Being part of an unfolding story: togetherness in everyday occupations when ageing. Scand J Occup Ther.

[53] Vik K, Eide AH (2014). Evaluation of participation in occupations of older adults receiving home-based services. Br J Occup Ther.

[54] Zingmark M, Evertsson B, Haak M (2020). Characteristics of occupational therapy and physiotherapy within the context of reablement in Swedish municipalities: A national survey. Health Soc Care Community.

[55] Bernhardt J, Hayward KS, Kwakkel G, Ward NS, Wolf SL, Borschmann K (2017). Agreed definitions and a shared vision for new standards in stroke recovery research: the stroke recovery and rehabilitation roundtable taskforce. Int J Stroke.

[56] Norlander A, Iwarsson S, Jönsson A-C, Lindgren A, Månsson Lexell E. Participation in social and leisure activities while re-constructing the self: understanding strategies used by stroke survivors from a long-term perspective. Disabil Rehabil. 2021:1–9. 10.1080/09638288.2021.1900418. 10.1080/09638288.2021.1900418 33779458

[57] Taule T, Strand LI, Skouen JS, Råheim M (2015). Striving for a life worth living: stroke survivors' experiences of home rehabilitation. Scand J Caring Sci.

[58] Zingmark M, Kylén M. Feasibility of a reablement-program in a Swedish municipality. Scand J Occup. Ther. 2022:1–12. 10.1080/11038128.2022.2089229. 10.1080/11038128.2022.2089229 35771642

